# Spread of the Specific Diseases—Paroxysmal Fevers—Marsh Fever Produced by Obstruction of the Outlets of Subsoil Water

**Published:** 1873-11

**Authors:** 


ARTICLE V.—*Spread of the Specific Diseases—Paroxysmal Fevers —Marsh Fever produced by Obstruction of the Outlets of Subsoil Water.*

The following account by Dr. Derby,* of Massachusetts, will be found of interest.

The uneven surface of Massachusetts has allowed great resort to water-power, and thus dams and reservoirs have been formed which obstruct flow, and have raised the water level.

The reservoir, also, which is often constructed to supply water for the wheel, is a receiver of many impurities, and purifies itself little by movement, or if the reservoir is emptied, a foul bottom is exposed to the sun.

Formerly, indigenous intermittents in Massachusetts were very rare, but when they occurred they were often referred to the mill-dams causing obstruction, and producing wet, marshy soil. In 1828 a dam thus constructed on the Housatonic caused such a development of intermittent fever that the people obtained legal authority to remove the dam, on the ground of the injury to the public health. At the present time the exemption from intermittent fever is far less complete than it was formerly. "Twenty-five years ago it was taught in our medical schools," says Dr. Derby, "that intermittent fever was a disease almost unknown in Massachusetts, except when contracted in other places. * * * * The exemption of the people of this State from periodic fever

* "Third Report (1872) of the State Board of Health of Massachusetts." Boston: 1872.

seems to be far less complete at the present time. Intermittents have been observed in Chelsea, and the history of the cases carefully investigated, and their local origin proved, on lands reclaimed from a salt-marsh, and now covered with houses. Cases have been seen during the past year clearly originating in Boston. They have been reported of late years in various parts of the State, along the banks of streams in low lands, and also in the immediate vicinity of tide-water, and occurring in persons who had never lived elsewhere.

"Physicians have observed that in the treatment of typhoid or enteric fever, the number of cases in which quinine is found useful, not simply as a tonic, but as a febrifuge, has become very considerable.

"In parts of Connecticut, intermittent and even remittent fevers are now not only frequent, but severe; and the gradual enlargement of the area of malarious influence, from the latitude of Long Island Sound toward the southern border of Massachusetts, is a fact to which we may well give heed."

The cause of this extension is to a considerable extent fresh-water obstruction, and several examples are given; but there is in addition another form of water obstruction, which, says Dr. Derby, "is full of danger to the public health, and is counterbalanced by no commensurate claims to economic value. If the fresh-water obstructions need to be watched, some of the salt-water obstructions need to be demolished. If the river-dams and reservoirs are liable, when used for the single object of profit, to render public health less secure, the tide-mills in crowded neighborhoods are nuisances which should be abated."

The rise and fall of the tide has been used as a motive power in all convenient situations from the earliest settlement of the country. Of late years tide-mills have not been found profitable, but the dams remain, and in most cases their water is still used. When first formed, there were seldom any houses near them, but now there is a crowded population close to them. In the case of the "Cross-dam" at Boston, about 70 acres were kept filled in 1871 by stagnant salt water, for the sake of the power thus obtained for a grist mill. This affected the health and comfort of at least 200,000 persons. * * * * A case which occurred at Natal, and which was related to me by Dr. Cattell, of the 10th Hussars, gives another interesting instance of an accidental obstruction acting somewhat in the same way. I give Dr. Cattell's memorandum in his own words:

" Fever of a severe and unusual type suddenly appeared on the coast northward of the Unguini river, and as a vessel had recently put into D'Urban, from Port Louis, disabled with Mauritius fever, the sick crew being landed at the Civil Hospital, a panic induced the Government to send down a Commission of Inquiry.

" Along a line of some five miles, a low hill range encloses a narrow belt of lagoons, themselves separated seaward by a low spit of sand, often in mounds, held together by juniper and iceplant, and pervious below to the water. To the south this range abuts on the Unguini, a large river; here, as it meets the sandy shore, sinking through and becoming shallow, having therefore a variable mouth, which changes after great floods. Six months previously, before the present hot season, an unusual rainfall, over a wide area near its sources, had brought down a large volume of water, destroying the bridge, and carrying trees, Kaffir houses, much soil, and a large mass of canes, refuse, manure, etc., from its banks and the low adjoining plantations.

" Previously opening near the lagoons, the river had now cut southward along the sandy shore and opened 600 yards or more further down, so that the seaward boundary of the lagoons extended right across its former mouth. The flow, instead of being toward the lagoons, was now directly away, and these were therefore not overflowed as deeply as formerly. The entrance to them from the river was found closed almost entirely by masses of vegetable matter, trees, canes, and canefield refuse, matted together to a depth of from four to six feet, lying always in the water, but now entirely submerged. All along the lagoons similar matter had been carried by the flow-water, and left partially submerged.

" Along the seaward face of the ridge, generally high up, houses were dotted, in every one of which scarcely any who remained had escaped the fever, which was most severe above the large mass of decaying matter near the old mouth, which affected every house to the leeward of the lagoons, but extended no further, and did not affect residents on the further (landward) side of the ridge. Adults and children, Kaffirs and Coolies, alike suffered. There were two local predisposing causes in operation, such as swampy ground and brackish water, which causes, however, had previously been quiescent." * * * "I believe they might all be classed as ague of a severe type and as ordinary remittent." * * * *

"Some months later, fever of a different type appeared, also suddenly, in a small village at an extremity of D'Urban Bay." * * * * "Here, at low tide, the water retreats from 500 to 600 feet, leaving alluvium partly sheltered by mangroves. For some time the night-soil of the neighboring town of D'Urban, collected from boxes, had been carted and deposited here, not very far above the level ground on the slope." * * * "The type was enteric, with delirium, and considerable abdominal irritation, showing marked intermissions, as do most acute cases in this neighborhood, especially fevers." * * * "There seems no doubt that here the fever was due almost entirely to animal poison; that the sewage matter deposited on soil whose natural outfall was interrupted by salt embankments, sufficiently accounted for the outbreak which some supposed arose from effluvium of these novel, unpopular salt-works, or from tidal alluvium, or from mangroves, which had existed before the houses were built, and which lined the shore along the township in the immediate vicinity of streets hitherto without suspicion."

